# Active Polysaccharide Films Incorporating *Cannabis sativa* Flower Extract for Extending the Shelf Life of Freeze-Dried Berries

**DOI:** 10.3390/molecules31030443

**Published:** 2026-01-27

**Authors:** Renata Dobrucka, Elżbieta Studzińska-Sroka, Magdalena Paczkowska-Walendowska, Judyta Cielecka-Piontek, Małgorzata Gumienna, Małgorzata Lasik-Kurdyś, Marcin Szymański

**Affiliations:** 1 Department of Non-Food Products Quality and Packaging Development, Institute of Quality Science, Poznan University of Economics and Business, al. Niepodległości 10, 61-875 Poznan, Poland; 2 Department of Pharmacognosy and Biomaterials, Poznan University of Medical Sciences, Rokietnicka 3, 60-806 Poznan, Poland; 3 Faculty of Food Science and Nutrition, Poznan University of Life Sciences, Wojska Polskiego 31, 60-624 Poznan, Poland; 4 Center for Advanced Technologies, Adam Mickiewicz University in Poznan, Uniwersytetu Poznańskiego 10, 61-614 Poznan, Poland

**Keywords:** polysaccharide films, bioactive compounds, freeze-dried berries, active packaging

## Abstract

In this study, films based on polysaccharides with *C. sativa* flower extract were prepared for selected freeze-dried fruits: raspberry (*Rubus idaeus* L.) and blueberry (*Vaccinium corymbosum* L.). The extract used affected the barrier and mechanical properties of the film. The elongation values of the film ranged from 32.5 ± 8.6 [%] (for sample 0) to 44.8 ± 8.2 [%] (for sample 4.0 F). The addition of the extract resulted in an increase in polyphenol content, proportional to the quantity of extract used. Spearman’s rank correlation analysis showed particularly strong correlations between colour indices (L*, a*, b*) and parameters describing antioxidant activity. The use of *C. sativa* flower extract in the polysaccharide matrix reduced the degradation of bioactive compounds during the storage of packaged fruit. In all cases of stored raspberries, a decrease in the number of moulds and yeasts was observed after 2 and 8 weeks. The greatest reduction in moulds and yeasts was recorded for the 4.0 F film (from 0.86 to 0.64 log cfu/g). In the case of blueberries, the total number of bacteria before storage was 2.52 log cfu/g, while after 8 weeks of storage in 4.0 F, this number significantly decreased to 2.28 log cfu/g. As in the case of raspberries, a reduction in mould and yeast was observed, with concentrations falling from an initial value of 0.89 to 0.67 log cfu/g after 8 weeks of storage at 4.0 F.

## 1. Introduction

Fruit is a type of food that plays a very important role in human nutrition. It is a rich source of vitamins, fibre and minerals. It also contains antioxidants that support the natural defence mechanisms of human cells. Due to their very low energy content, they are recommended by dieticians to people who want to maintain a healthy body weight [1]. Raspberries and blueberries, which are available all year round, are very popular. Raspberries are considered a noble fruit due to their unique taste and elemental composition. They are characterised by demanding growing conditions, high production costs and perishability. Fresh fruit contains over 85% water and a significant amount of fibre, making raspberries ideal for people trying to lose weight and lower their cholesterol, as well as for type 2 diabetics. In Japan, red raspberry ketones are used as an ingredient in dietary supplements to aid weight loss. Raspberries also contain many antioxidants, such as vitamin C and anthocyanins, gallic acid and quercetin. Blueberries, on the other hand, contain vitamins A, B1, B2, and B3, as well as phosphorus, potassium, calcium, sodium, folic acid, and phytoestrogens. In addition, they are characterised by high antioxidant activity, resulting from the value of polyphenols, especially anthocyanins [2]. The health benefits of blueberries have been known for ages. The fruit is used to help prevent conditions like lifestyle diseases and heart problems. Raspberries have been used for thousands of years for nutrition and medicine [3].

This fruit is most often found in freeze-dried form. This is one of the methods of preserving food so that these fruits are available all year round, not just during the harvest season. The way freeze-dried fruits are stored is very important in this regard. Recently, consumers have been increasingly reaching for this type of healthy snack, which provides a specific dose of vitamins and nutrients. The freezing process preceding freeze-drying practically stops chemical and biochemical changes. As a result, the freeze-dried product has a taste, smell and colour similar to fresh fruit. As much as 98% of all nutrients are retained. As a result, no artificial additives such as preservatives, colourings, flavour enhancers or synthetic vitamins need to be added to the product. In turn, the low temperature of the process and the absence of air in the freeze-drying chamber allow thermolabile and easily oxidisable compounds to be preserved. In a water-free environment, no bacteria can develop—all decomposition and spoilage processes are completely inhibited. On the other hand, the low water content of freeze-dried products, often at the level of 2–3%, means that there is no monolayer to act as a barrier to oxygen contact, which, combined with high porosity, creates good conditions for the oxidation of freeze-dried components. Furthermore, exposure to temperature and light changes during transport requires the use of appropriate packaging material for this type of product [4].

Nowadays, people are looking for materials that meet packaging requirements. Every initiative shows the global trend towards reducing plastic waste to lower the impact on human health and the environment [5]. It is currently taking a number of measures to combat plastic pollution and accelerate the transition to a circular economy. Currently, the European Union is taking a number of measures to combat plastic pollution and accelerate the transition to a circular plastics economy. Several of the proposed measures specifically address plastics and packaging waste, with the ultimate goal of achieving climate neutrality by 2050 [6]. Not all recycled materials can be used for food packaging. Biopolymer-based materials are very interesting from the perspective of sustainable development and consumer safety. They are a viable substitute for traditional materials due to their inherent biodegradability, non-toxicity, and biocompatibility. They have the ability to significantly mitigate the environmental impact associated with energy consumption and carbon dioxide emissions associated with conventional packaging [7,8]. Consequently, materials based on polysaccharides are become increasingly valuable. Polysaccharides, ones of the most common biopolymers in nature, are long-chain macromolecules formed by linking monosaccharides with glycosidic bonds [9,10]. Often, active additives such as antibacterial or antioxidant agents need to be incorporated in order for them to gain active functions. Oxidation and microbial contamination are the main causes of food spoilage, and substances with antioxidant and antibacterial properties are added to films in order to produce active films [11,12].

This study developed packaging materials based on plant-derived polysaccharides, namely citrus pectin, gellan gum, and *Cannabis sativa* flower extract. Gellan gum (E418) is a polysaccharide produced by microbiological methods. It has gelling and stabilising properties. It is used in the production of medicines, cosmetics and food products. Pectin (E440) is an anionic, amorphous and non-toxic polymer naturally occurring in plant cell walls, mainly in various fruit and vegetable skins and pulp. Most commercial pectin comes from apple pomace and citrus peel [13]. The WHO/FAO Expert Committee has classified both polysaccharides as food additives. Pectin exhibits some antioxidant activity, which is directly related to the presence of hydroxyl groups. Additionally, it could therefore be expected that hydrogen bonding interactions between the hydroxyl or carboxyl groups of pectin and the obtained flower extract from *Cannabis sativa* could occur, contributing to the formation of a density and compact matrix with improved properties [5,14,15].

*Cannabis sativa* flower is a cluster of flowers that grows at the top of the plant. One of the most characteristic features distinctive to cannabis is the presence of specific compounds called cannabinoids, which, together with terpenes, are produced in specialised epidermal glands (secretory hairs) located throughout the plant, mainly in the apical parts of the panicles [16]. Cannabinoids, as secondary metabolites, enable plants to interact with their environment and contribute to their survival. Canabinoid properties are used in the treatment of many diseases, such as glaucoma, asthma, malaria, hypertension, and constipation [17]. Besides terpenes, cannabis plants also have polyphenols. The most important polyphenols in this plant are flavonoids, stilbenes, and lignans. Most of the flavonoids found in cannabis so far are flavones and flavonols. The most important flavonoid aglycones include apigenin, luteolin, quercetin and kaempferol [18]. Due to the presence of so many active ingredients, flower extract was used for the polysaccharide matrix. To the best of our knowledge, this study is the first to use films with added *C. sativa* flower extract. Fruits (raspberries and blueberries) were also selected for the experiment, as they have rarely been used by other researcher to analyse microbiological stability during storage.

## 2. Results and Discussion

### 2.1. GC-MS Analysis of Cannabis sativa Flower Extract

**Figure 1:**
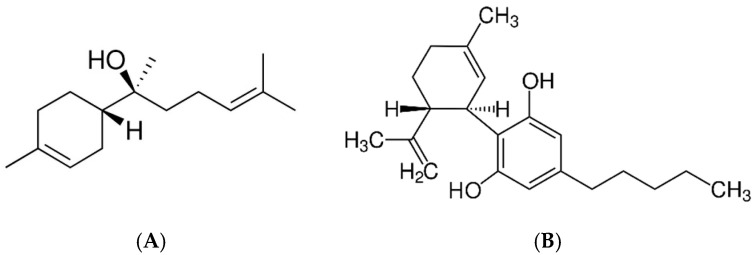

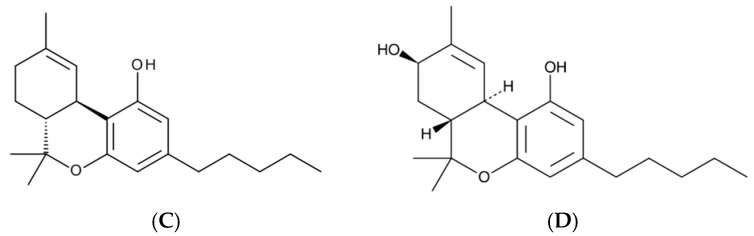
The main compounds found in methanol extract from C. *sativa* flowers: (**A**) α-bisabolol, (**B**) cannabidiol, (**C**) Δ^9^-tetrahydrocannabinol and (**D**) 8-ß hydroxy-d 9-tetrahydrocannabinol.

Table 1 presents the GC–MS analysis of the ethanolic extract, indicating the presence of several compounds characteristic of *Cannabis sativa* flowers. The highest proportions in the analysed extract were represented by cannabidiol (86.95%), Δ^9^-tetrahydrocannabinol (2.02%), 8-β-hydroxy-Δ^9^-tetrahydrocannabinol (2.64%), and α-bisabolol (1.31%) (Figure 1). One of the identified components was α-bisabolol, which, according to literature data, exhibits anti-inflammatory, anti-irritant, antibacterial, antifungal, anticancer, anti-infective, anticholinesterase, and anti-allergic properties. This compound is widely used in products intended for the care of sensitive skin [19] and has been recognized as safe (GRAS), which has contributed to its use as an active ingredient in various commercial formulations [20]. Cannabidiol is a 21-carbon terpenophenolic compound that interacts with multiple molecular targets [21]. Studies indicate its broad spectrum of pharmacological activity, including analgesic, anti-inflammatory, antiepileptic, and anxiolytic effects, which confirms its therapeutic potential in the treatment of neurodegenerative diseases and neuropsychiatric disorders [22]. Its antioxidant, anti-rheumatic, and anticancer properties have also been reported [23]. Δ^9^-tetrahydrocannabinol is one of the biologically active compounds naturally occurring in *C. sativa* inflorescences. It has been used in the treatment of chemotherapy-induced nausea and vomiting, AIDS-related wasting syndrome, anorexia, and post-traumatic stress disorder (PTSD) [24,25]. It should be emphasized that the present study had a model character and focused on the analysis of the behaviour and presence of bioactive compounds within the polymer matrix. The presented results provide a basis for further application-oriented studies, which may aim to evaluate the migration of active compounds, their stability, safety, and regulatory compliance in the context of potential practical applications.

### 2.2. HPLC Analysis and Dissolution Studies

**Figure 2:**
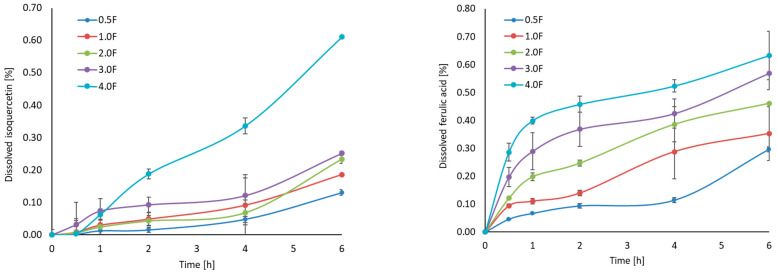

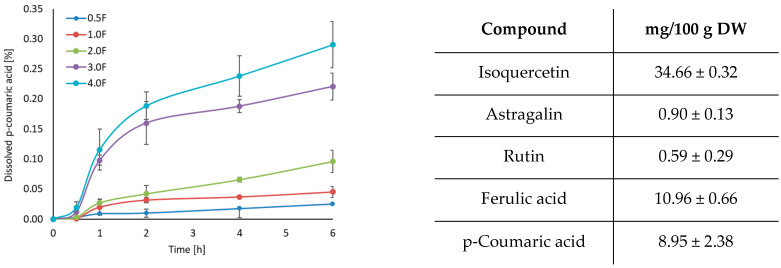
Dissolution profiles (isoquercetin, ferulic acid, p-coumaric acid) and results of HPLC analysis.

HPLC analysis of the extract confirmed the presence of several biologically active phenolic compounds, including isoquercetin (34.66 ± 0.32 mg/100 g DW), ferulic acid (10.96 ± 0.66 mg/100 g DW), and p-coumaric acid (8.95 ± 2.38 mg/100 g DW). The release profiles of these compounds varied between formulations (Figure 2). Among the tested samples, the 4 F film demonstrated the highest release of isoquercetin, reaching approximately 0.6% after 6 h. This trend was also observed for ferulic and p-coumaric acids, with the most effective release being observed in the 4.0 F film. This suggests that increasing the concentration of the active extract in the film matrix facilitates diffusion into the aqueous environment. Interestingly, ferulic acid showed the fastest and highest total release, which can be attributed to its higher polarity and better water solubility than other compounds [26]. These results indicate that the amount of substance released depends on the composition of the matrix and the physicochemical properties of the individual compounds. From the point of view of the food industry, the ability to gradually release polyphenolic components from biopolymer-based films may support their potential application. This type of packaging can provide prolonged protection for the packaged products. Therefore, the polyphenols will not only be effective for a short time, but their activity will be sustained over a longer period, which increases their effectiveness and thus extends the quality of the packaged product—in our case, freeze-dried fruit. Sudden hydration could lead to rapid depletion of polyphenols and, consequently, loss of effectiveness. Therefore, the release results for our films are very favourable, as they allow for longer, more stable and safer action of the bioactive substances contained in the cannabis flower.

### 2.3. FTIR of Prepared Hydrocolloid-Based Films Polysaccharides

**Figure 3:**
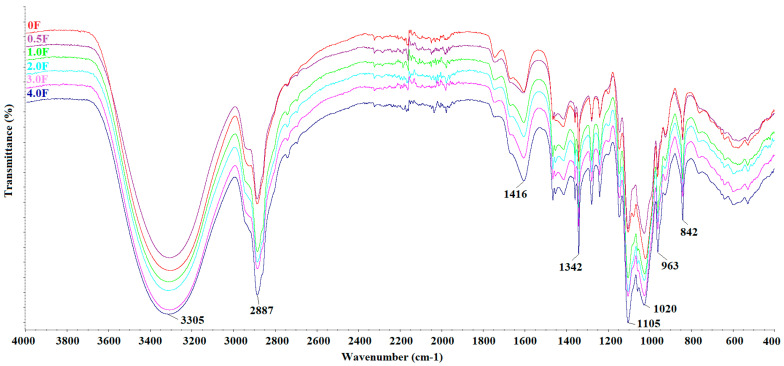
FTIR spectra of films: 0 F, 0.5 F, 1.0 F, 2.0 F, 3.0 F and 4.0 F.

**Figure 4:**
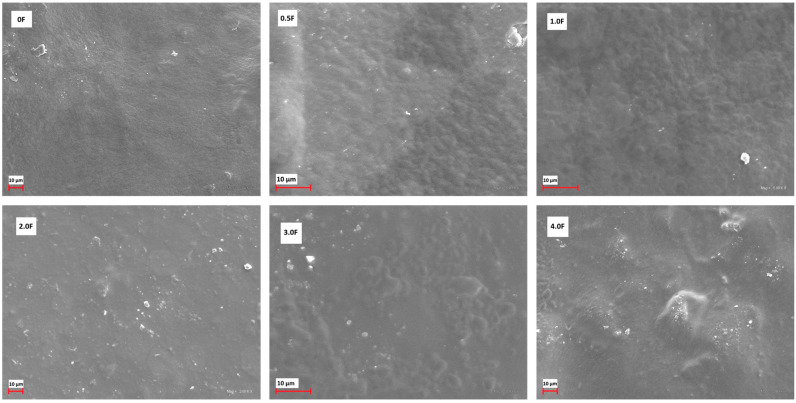
SEM images for active films: 0 F, 0.5 F, 1.0 F, 2.0 F, 3.0 F and 4.0 F.

Figure 3 shows the FTIR spectra of all fabricated films. The following bands were observed: 3304 cm^−1^, 2887 cm^−1^, 1416 cm^−1^, 1342 cm^−1^, 1104 cm^−1^, 1020 cm^−1^, 962 cm^−1^, and 842 cm^−1^. The broad band at 3304 cm^−1^ can be attributed to O–H and C–H stretching vibrations, while the band at 2887 cm^−1^ corresponds to C–H stretching in methyl and methylene groups. The band at 1416 cm^−1^ is associated with C–OH stretching, and the bands at 1104 cm^−1^, 1020 cm^−1^, 962 cm^−1^, and 842 cm^−1^ are typical for C–O–C and C=H vibrations [27,28]. The FTIR spectra of all films are similar, with no new bands or significant shifts observed. Differences in the intensity of certain bands were noted depending on the amount of extract used, which may reflect changes in the film composition. These observations confirm the presence of the expected functional groups in the polysaccharide films and the incorporation of the cannabis flower extract [29,30]. Surface morphology analysis of the films using SEM (scale 10 µm, Figure 4) revealed smooth and homogeneous surfaces for all samples, regardless of the extract concentration. These features indicate a continuous and coherent polysaccharide matrix in which the extract is evenly distributed. The homogeneous film surface may suggest a favourable distribution of active compounds within the matrix, which is a desirable property for potential application in functional packaging materials.

### 2.4. Study of Hydrocolloid-Based Films Polysaccharides (Mechanical, Barrier, Density, Swelling Index)

**Figure 5:**
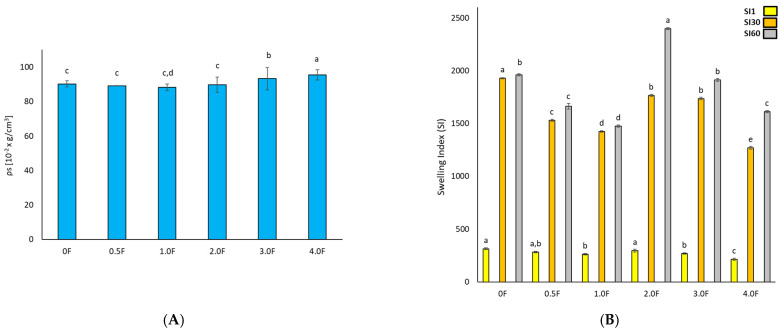

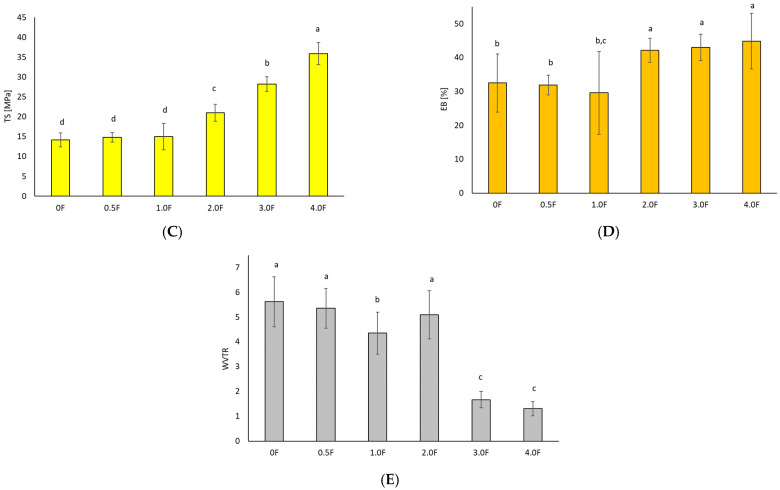
Test results for hydrocolloid-based films polysaccharides (**A**) density, (**B**) swelling index, (**C**) tensile strength, (**D**) elongation at break, and (**E**) WVTR. The letters (a, b, c, …) next to the mean values indicate statistical significance of differences between groups, determined by a post-hoc test (Tukey).

Figure 5 shows the results of the film tests (A) density, (B) swelling index, (C) tensile strength, (D) elongation at break, and (E) WVTR. It can be clearly observed that the density of the film increases with the increase in the extract additive. The greatest increase in density was observed in films 3.0 F and 4.0 F, i.e., 93.25 ± 6.48 [10^−2^ g/cm^3^] and 95.52 ± 3.00 [10^−2^ g/cm^3^]. This is the result of greater cross-linking due to the interaction of active compounds in the extract and the polysaccharide matrix [31]. The higher the extract content, the higher the density of the obtained films, which had a direct impact on their physical properties. A decrease in the swelling coefficient was also observed with an increase in the extract. The swelling index measures the maximum water absorption ability of the film before its structural damage, reflecting its potential usefulness as a water transmission material. Swelling behaviour is largely dependent on the extent of intermolecular interactions in polymer chains [32].

The highest swelling index value was observed for sample 0 F (2614.98 ± 8.84). The addition of the extract to the polysaccharide matrices resulted in a decrease in the swelling coefficient. For the 1.0 F, 2.0 F, 3.0 F and 4.0 F films, the values were 2572.44 ± 17.73, 2416.54 ± 7.22, 1956.51 ± 9.11, and 1655.29 ± 5.77, respectively. This is the result of hydrogen bonds between the chains of the polysaccharides used and the polyphenols contained in the extract [33]. Therefore, films with the extract added are characterised by greater cross-linking and consequently less free space for liquid absorption, which is often a desirable feature in food films. The addition of the extract thus made the packaging material more hydrophobic and less able to absorb water.

In the quality assessment of the developed materials, it was necessary to analyse their mechanical and barrier properties to determine whether they are able to withstand external stresses and maintain their integrity as a barrier for the environment and packaged food [34]. By measuring these properties, it is possible to provide a prediction of how the material will react under different food processing conditions and compare it with conventional packaging. According to research, films made from polysaccharides have similar tensile strength (TS) values comparable to synthetic polymers [35,36]. Furthermore, the mechanical properties are related to the film structure, where a compact film structure provided relatively better fracture toughness [37]. Additives to the polymer matrix are also important. The interaction of polyphenolic compounds with film components can improve its compactness and mechanical tensile strength [38].

In this study, an increase in tensile strength was observed (*p* < 0.05) with an increase in the amount of extract, which was related to the polyphenol content present in the in-florescence. The elongation values ranged from 32.5 ± 8.6% (sample 0) to 44.8 ± 8.2% (sample 4.0 F). This phenomenon may be associated with changes in the polymer network structure induced by the incorporation of the extract, potentially affecting chain mobility and intermolecular interactions [39]. However, it should be emphasized that this is a hypothesis that has not been confirmed by direct molecular-level investigations. Different trends have been reported in the literature for other biopolymer systems. In the case of sericin–pectin films, a decrease in elongation at break was observed with increasing additive content [40,41,42,43], whereas the addition of neem leaf extract to pectin–chitosan films led to a reduction in tensile strength [44]. These differences indicate that the mechanical response of the material strongly depends on the type of polymer matrix and the nature of the incorporated bioactive compounds. In the studied system, compounds identified by GC–MS analysis may have contributed to the observed increase in film flexibility; however, their precise role and mode of interaction with the pectin–gellan matrix require further investigation. Water vapour transmission rate (WVTR) is one of the key parameters in the selection of packaging materials, as it affects moisture transfer and the shelf life of stored products [45,46]. In the present study, the addition of the extract was associated with a decrease in WVTR values, which ranged from 5.62 ± 1.01 g/m^2^·d (sample 0 F) to 1.30 ± 0.29 g/m^2^·d (sample 4 F). However, it should be noted that these values are exceptionally low for hydrophilic pectin- and gellan-based films, particularly in light of the high water absorption observed. The reduction in WVTR may result from changes in film microstructure or enhanced interactions within the polymer matrix induced by the presence of the extract. Therefore, although the obtained results indicate promising barrier properties, further studies are necessary to confirm the mechanisms responsible for the low WVTR values and to clarify the apparent contradiction between low water vapour permeability and high water uptake.

### 2.5. Testing of TPC, TFC, Antioxidant Activity of Hydrocolloid-Based Films, Polysaccharides and Extract (DPPH, ABTS, CUPRAC, Cu^2+^ Chelating) and Dissolution Studies

**Figure 6:**
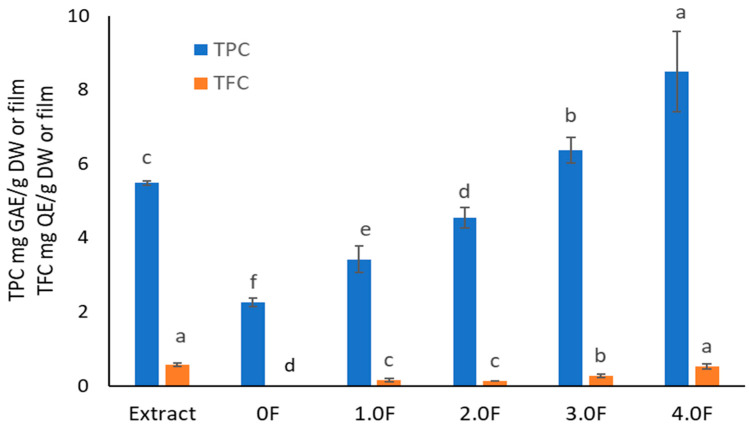
The study of TPC and TFC levels in extract and polysaccharide films. The letters (a, b, c, …) next to the mean values indicate statistical significance of differences between groups, determined by a post-hoc test (Tukey).

Plant extracts represent a valuable source of compounds with antioxidant properties, which allows them to play a significant role in counteracting oxidative stress [47]. This phenomenon leads to the degradation of components in food, pharmaceuticals, and cosmetics, ultimately reducing their stability and quality [48]. Consequently, there is growing interest in the use of natural antioxidants in packaging materials designed to protect products particularly sensitive to pro-oxidative factors. The concept of so-called active packaging thus represents a modern research direction focused on enhancing the functionality and utility of packaging materials. It is well established that the antioxidant activity of plant extracts is primarily associated with the content of secondary metabolites, particularly polyphenolic compounds. Therefore, an analysis was conducted on an extract obtained from *C. sativa* flowers, with determination of total phenolic content (TPC, expressed in mg gallic acid equivalents per gram of dry weight, mg GAE/g DW) and total flavonoid content (TFC, expressed in mg quercetin equivalents, mg QE/g DW)—both of which are considered key contributors to the antioxidant activity of plant-derived materials (Figure 6). Spectrophotometric analyses showed that the *C. sativa* flower extract contained TPC at a level of 5.49 ± 0.06 mg GAE/g DW and TFC of 0.57 ± 0.05 mg QE/g DW. These results are consistent with previous findings regarding the phenolic composition of this species [49]. However, compared to TPC values commonly reported for other plant extracts (e.g., green tea, rosemary), the result can be considered relatively low [50,51]. Of particular note is the relatively high proportion of flavonoids, accounting for approximately 10% of total phenolics—similar observations have been previously reported in the literature [49]. This suggests that flavonoids may significantly contribute to the observed moderate biological activity of the extract. Flavonol-type compounds have been reported in extracts from various *C. sativa* flower cultivars, according to available literature [52]. Among phenolic acids, the content of p-coumaric and ferulic acids in the extract was determined. Studies on TPC and TFC levels in packaging film samples modified with the extract demonstrated that the addition of the plant extract during processing led to an increase in polyphenol content—proportional to the amount of extract used. For sample 0.5 F, TPC was 2.98 ± 0.17 mg GAE/g film, while in sample 4 F it reached 8.50 ± 1.08 mg GAE/g film. Likewise, TFC increased from 0.13 ± 0.01 mg QE/g film to 0.52 ± 0.08 mg QE/g film. This increase in bioactive components may positively influence the moderate antioxidant properties of the material.

**Figure 7:**
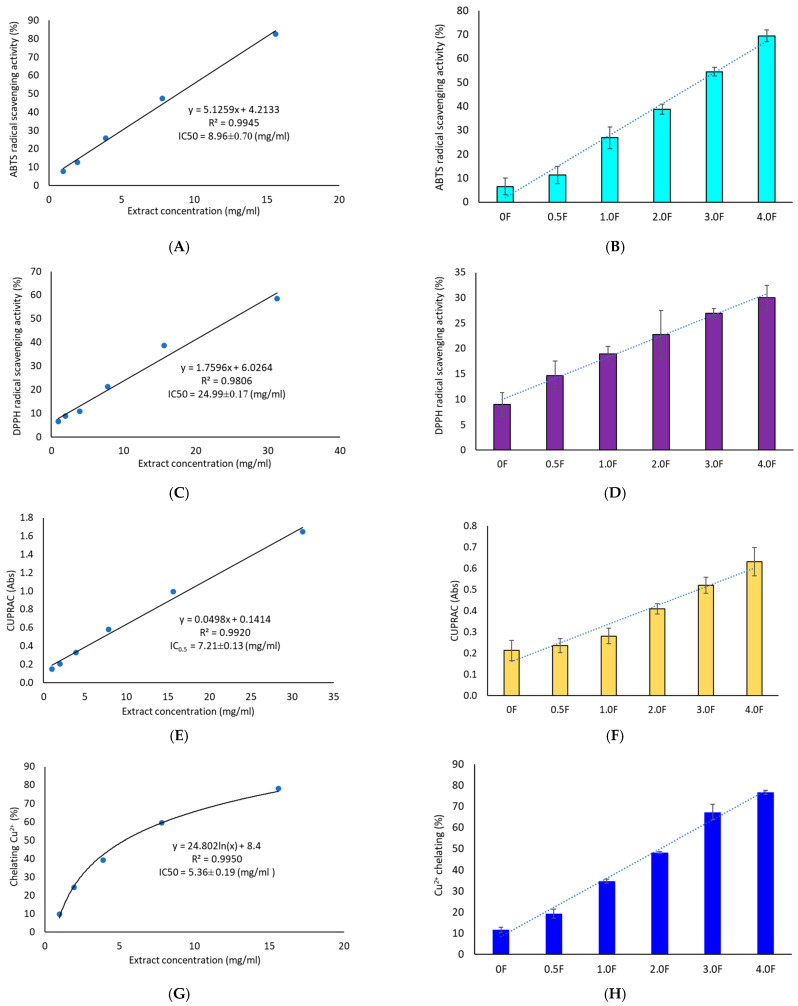
Antioxidant activity study of extracts (**A**,**C**,**E**,**G**) and polysaccharide films (**B**,**D**,**F**,**H**) using ABTS, DPPH, CUPRAC and metal ion chelation (Cu^2+^) methods.

The antioxidant activity of the extract was evaluated using four methods: DPPH, ABTS, CUPRAC, and metal ion (Cu^2+^) chelation assay. All tests confirmed the extract’s ability to neutralize free radicals and transition metal ions, with higher extract content in the films corresponding to higher moderate antioxidant activity (IC50 = 24.99 ± 0.17 mg/mL—DPPH, IC50 = 8.69 ± 0.70 mg/mL—ABTS, IC50 = 7.21 ± 0.13 mg/mL—CUPRAC, IC50 = 5.36 ± 0.19 mg/mL—Cu^2+^ chelation, Figure 7). This study demonstrates that *C. sativa* flower extract exerts a measurable effect on the antioxidant activity of films, which can serve as a basis for further application-oriented studies. According to the literature, the introduction of phenolic extracts into pectin/gelatin composite films enhances the antioxidant properties of pectin films [53]. Adilah et al. (2018) and Koirala et al. (2023) also obtained antioxidant films using mango peel extract, which played a significant role in the antioxidant activity of the film [54,55]. Similarly, Pirsa et al. (2024) reported that the addition of onion peel extract to CMC-based films significantly increased the films’ activity in terms of DPPH radical scavenging from 11 to 63% [54,55,56]. Biratu et al. (2024) observed in their studies the antioxidant properties of active films after the application of honey and propolis [57]. In a study conducted by Bhatia et al. (2024), an edible film composed of pectin and xanthan gum with the addition of grapefruit essential oil was developed [58]. It was found that the film has beneficial properties, enhancing moderate antioxidant activity, as evidenced by increased DPPH and ABTS values [58].

Importantly, literature data indicate that the DPPH assay is more sensitive to lipophilic compounds [59], whereas the ABTS assay is more responsive to polar substances [60]. The higher activity observed in the ABTS test therefore suggests that the moderate antioxidant activity of *C. sativa* extract may be mainly attributed to polar compounds. This conclusion is further supported by our results, as HPLC analysis detected a significant amount of flavonoid glycosides. Our findings are also consistent with the available literature [52], which extensively characterizes the phytochemical profile of *C. sativa* flower extracts and indicates that the proportion of polar compounds among the identified substances is higher than that of lipophilic ones. Chelation of transition metal ions such as copper (Cu^2+^) is a key mechanism of protection against oxidative stress triggered by the Fenton–Haber–Weiss reaction [61]. The extract’s ability to chelate Cu^2+^ ions (IC50 = 5.36 ± 0.19 mg/mL) appears promising for a natural-origin substance. For comparison, quercetin tested using the same method exhibited 79.47 ± 3.14% chelating ability at a concentration of 2.5 mg/mL [62]. Numerous studies also report a strong correlation between TPC/TFC values and results from CUPRAC and ABTS tests [63]. This trend was confirmed in the present study—an increase in antioxidant activity of the films was observed across all assays, accompanied by a clear increase in total phenolics, including flavonoids. This was also confirmed by a cluster analysis, which showed strong links between TPC/TFC and antioxidant activity measurements.

### 2.6. Testing the Colour Components (L, a, b) of Films Based on Hydrocolloid, Polysaccharides and Extract

**Figure 8:**
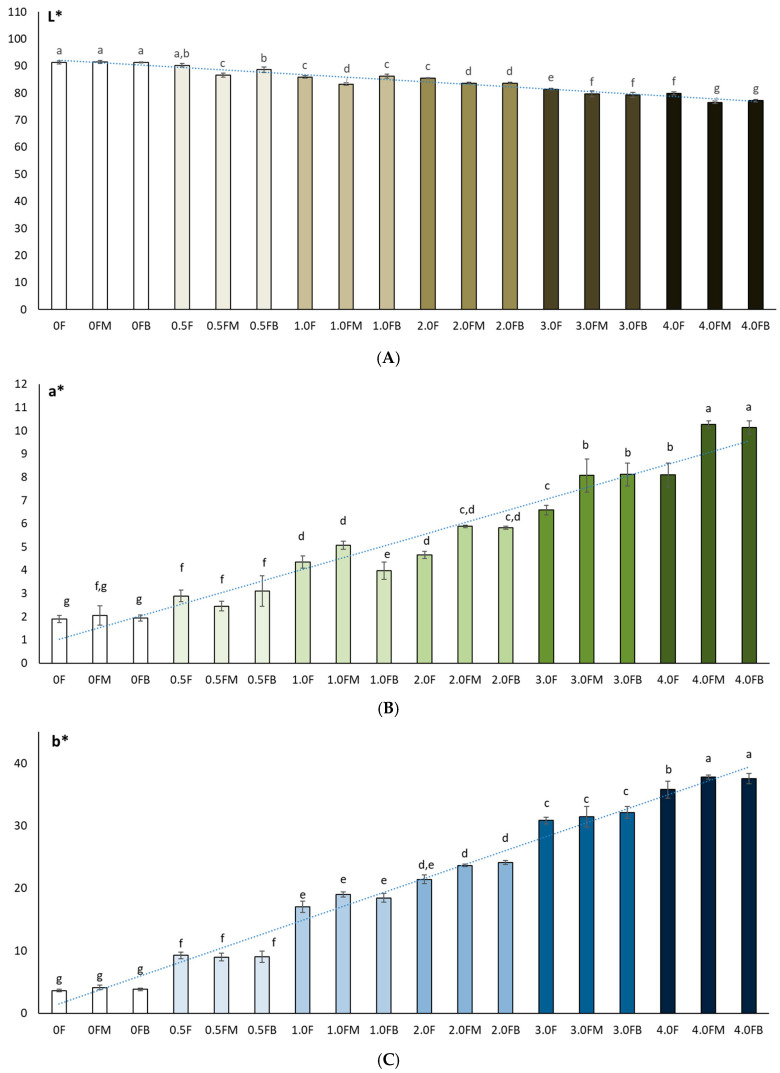
Colour measurements (L* (**A**), a* (**B**), b* (**C**)) of polysaccharide films before and after 8 weeks of fruit storage. The letters (a, b, c, …) next to the mean values indicate statistical significance of differences be-tween groups, determined by a post-hoc test (Tukey).

Colour plays a crucial role in the life cycle of both the packaged product and the packaging itself. Perceptions generated by packaging influence the general image of the product, which is why there is an increasing need to distinguish packaging from other products. Coloured films can also play a protective role, as they act as a barrier to light and reduce the oxidation of packaged products. In this study, it was observed that the matrix without the addition of extract, regardless of whether it was tested before or after fruit storage, was characterised by a high L* value (0 F = 91.33 ± 0.55; 0 FM = 91.44 ± 0.52; 0 FB = 91.27 ± 0.19), and thus a distinct brightness (Figure 8). The colour of the bars presented in the graphs reflects the increase in the concentration of the hemp flower extract used in the production of the film. These were higher brightness values than commercial pectin film (L* = 89.00) [64] and that presented by Çavdaroğlu et al. (2023), who reported an L* value of 77.27 for citrus pectin film [65]. In this study, the addition of extract caused a decrease in L* [65]. The increase in the content of extract and polyphenols in the obtained films was accompanied by a decrease in their brightness. Meanwhile, Hoque et al. (2024) observed that in the case of pectin/sodium alginate-based films with microcrystalline cellulose and geraniol, the addition of extract resulted in L* values [66]. Shankar and Rhim (2016) also noted an increase in L* values after adding 3.0% MCC to agar-based composite films [67]. Colour changes in the film therefore depend strongly on the chemical nature and composition of the added extracts. The film showed a higher decrease (*p* < 0.05) in L* and an increase in a* and b* with an increase in the amount of extract (polyphenol content) from the flowers. This was confirmed by Spearman’s rank correlation analysis. The correlation matrix (Figure 8) revealed a very high level of interdependence between colour indices (L*, a*, b*) and parameters describing antioxidant activity (TPC, DPPH, ABTS, CUPRAC, Cu^2+^ chelating capacity) [67].

### 2.7. Analysis of Changes in TPC and TEAC Content in Freeze-Dried Raspberry and Blueberry Fruits During Storage

**Figure 9:**
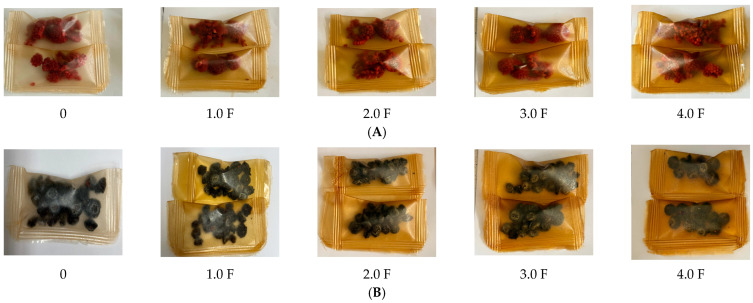
Images of raspberries (**A**) packaged in polysaccharide films: 0 F, 1.0 F, 2.0 F, 3.0 F, 4.0 F and blueberries (**B**) packaged in polysaccharide films: 0 F, 1.0 F, 2.0 F, 3.0 F, 4.0 F.

Phenolic compounds and their antioxidant activity play a key role in maintaining the quality of berries during storage. In this study, changes in the content of total polyphenols (TPC) and antioxidant activity (TEAC) in freeze-dried raspberry and blueberry fruits during 8 weeks of storage were analysed, taking into account the packaging film obtained with the addition of various concentrations of *C. sativa* flower extract (0 F–4.0 F). Figure 9 shows photographs of the resulting films with raspberries and blueberries packed inside them. Studies by Zielonka-Brzezicka et al. (2017) on the antioxidant properties of raspberry and blueberry fruits have shown that freeze-drying not only retains their natural properties but also affects their stability during storage [68]. According to studies, freeze-drying minimizes the loss of valuable bioactive substances; however, after a certain storage time, their activity may decrease, which is typically attributed to polyphenol oxidation and enzymatic reactions [69,70].

Changes in the content of polyphenols and antioxidant activity in freeze-dried raspberry and blueberry fruits may be the result of mutual interactions that occur during storage. The selection of parameters during the storage process, along with packaging properties, can help preserve the valuable properties of the raw material, which is crucial for maintaining its nutritional and sensory values for a longer period. As a result of the conducted analyses, it was found that regardless of the stored freeze-dried raspberry and blueberry fruits, after 8 weeks of storage, a decrease in the content of compounds with a polyphenolic character and their antioxidant activity was noted. However, in the case of blueberries, higher initial values of TPC and TEAC were noted and thus better stability during storage.

**Figure 10:**
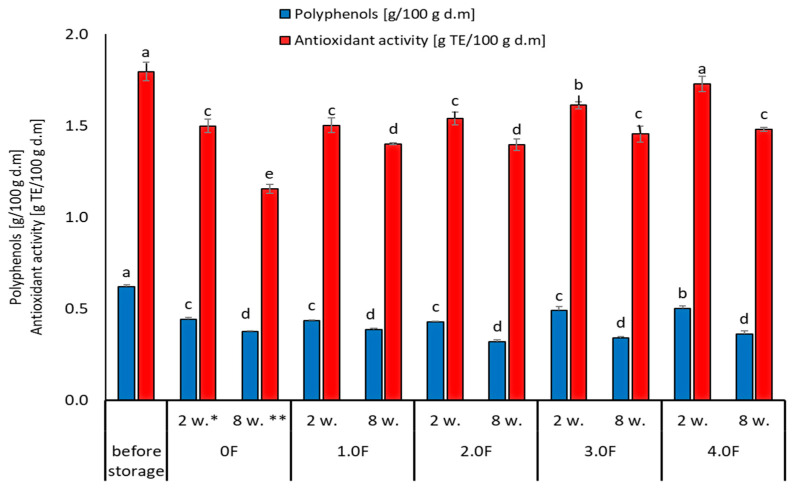
Changes in the content of phenolic compounds and antioxidant activity of raspberry during storage. *—2 weeks. **—8 weeks. The letters (a, b, c, …) next to the mean values indicate statistical significance of differences be-tween groups, determined by a post-hoc test (Tukey).

**Figure 11:**
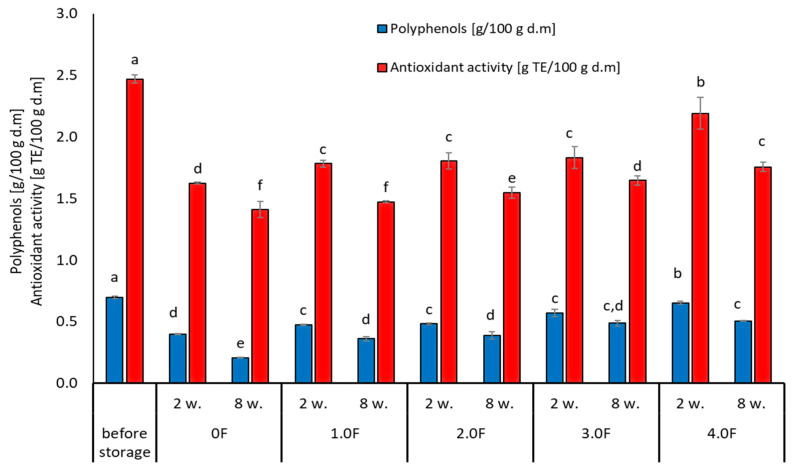
Changes in the content of phenolic compounds and antioxidant activity of blubbery during storage. The letters (a, b, c, …) next to the mean values indicate statistical significance of differences be-tween groups, determined by a post-hoc test (Tukey).

The greatest changes in the analysed parameters were observed in samples where the control variant of the film (0 F) was used, which suggests that the use of film with the addition of *C. sativa* flower extract (1.0 F–4.0 F) has an impact on the reduction in the degradation processes of bioactive compounds during storage. The 2.0 F–4.0 F variants used during storage tests are the most promising in terms of the preservation of biologically active compounds in freeze-dried raspberries and blueberries (Figure 10 and Figure 11). Moreover, it should be noted that the dynamics of changes in these compounds depended on the variant of the film used, which is visible in the case of 3.0 F, regardless of the fruit tested, which was characterized by better stability to TPC and TEAC after 2 and 8 weeks of storage.

Tseng and Zhao (2012) drew attention to the fluctuations in polyphenol levels during storage, noting that some compounds decreased by up to 40% in different types of berries [71]. In addition, the stability of the analysed compounds may be affected by the physical state of the berries after drying. Lavefve et al. (2020) and Syamaladevi et al. (2011) found that the kinetics of anthocyanin degradation in freeze-dried blueberries or raspberries followed a consistent kinetic model, showing both increases and decreases during storage, probably due to structural changes in the fruit matrix [72,73]. The packaging material and environmental storage conditions play an equally important role during the storage process. Zorić et al. (2015) found in their research that the stability of individual polyphenols in freeze-dried cherry fruits was significantly influenced by the materials used for packaging and storage conditions [74]. This leads to the conclusion that proper packaging can mitigate some of the polyphenol degradation processes and thus provide additional protection for maintaining the stable content of these compounds [74].

### 2.8. Study of the Colour of Freeze-Dried Raspberry (Rubus idaeus L.) and Blueberry (Vaccinium corymbosum L.) Fruit During Storage

**Figure 12:**
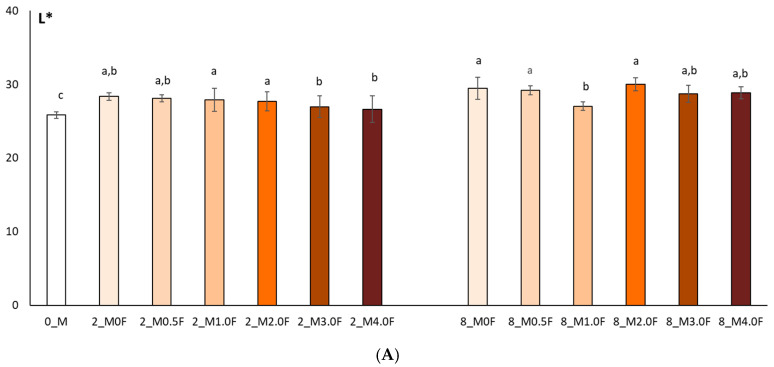

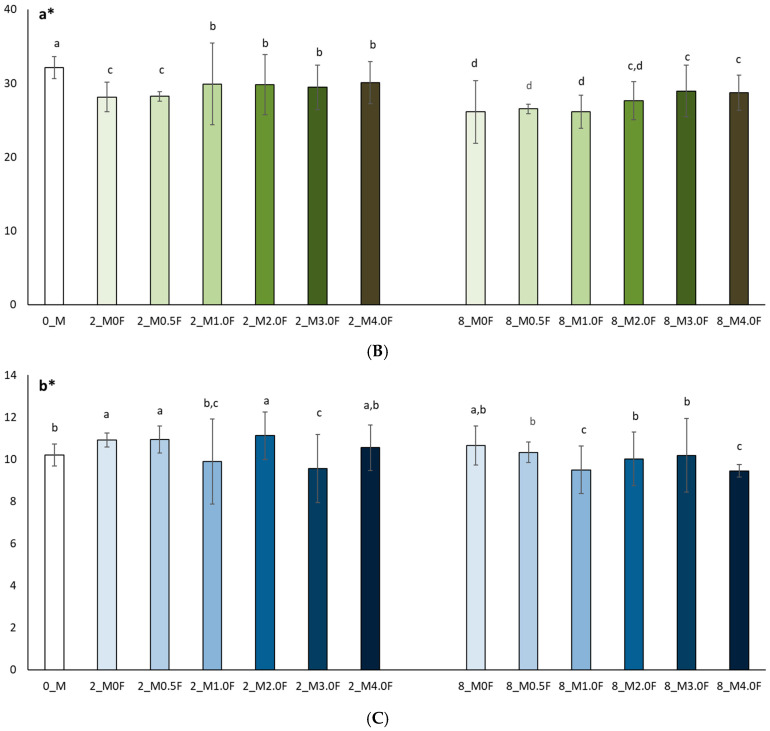
Colour test results (L* (**A**), a* (**B**), b* (**C**)) of raspberries after 0, 2 and 8 weeks of storage. The letters (a, b, c, …) next to the mean values indicate statistical significance of differences be-tween groups, determined by a post-hoc test (Tukey).

**Figure 13:**
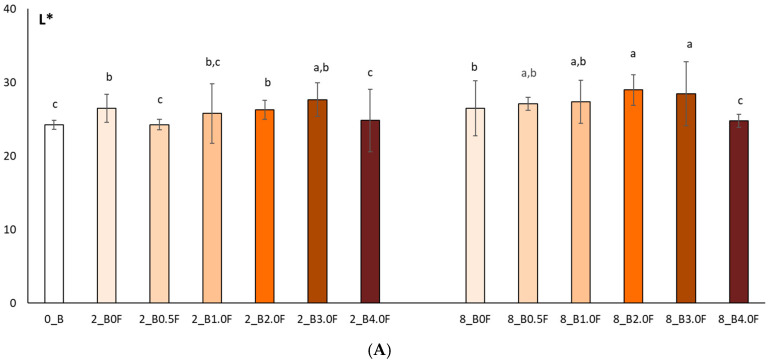

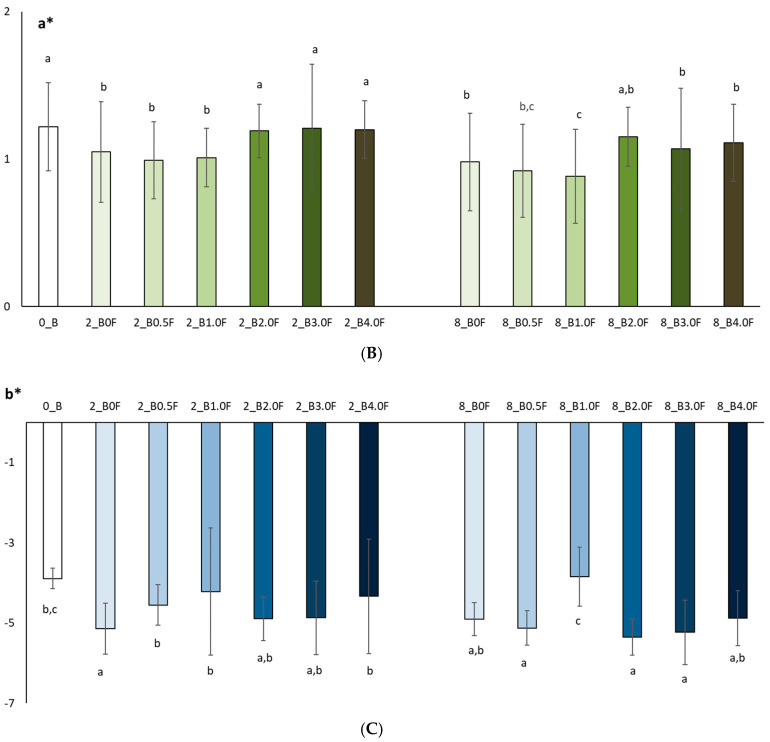
Colour test results (L* (**A**), a* (**B**), b* (**C**)) of blueberries after 0, 2 and 8 weeks of storage. The letters (a, b, c, …) next to the mean values indicate statistical significance of differences be-tween groups, determined by a post-hoc test (Tukey).

In this study, the colour of freeze-dried raspberry (*Rubus idaeus* L.) (Figure 12) and blueberry (*Vaccinium corymbosum* L.) (Figure 13) fruit was analysed at selected time points during storage (at 0, 2 and 8 weeks). The studies conducted on changes in polyphenol content and antioxidant activity in fruits stored in films with the addition of flower extract showed that with an increase in the amount of extract in the film, the antioxidant parameters of the fruits degraded more slowly, and, as a result, the stability of health-promoting compounds in such films was higher. *L parameter in the entire research series for both raspberries and blueberries increased slightly, which is inversely correlated with polyphenol content and antioxidant activity. The decrease in value *a suggests a loss of red colour in raspberries and blueberries, which may indicate the degradation of anthocyanins. This hypothesis is confirmed by the decrease in the amount of polyphenols determined in the fruit. The *b parameter of fruit colour did not show any statistically significant changes. The colour of the columns shown in the graphs in Figure 12 and Figure 13 reflects the increase in the concentration of hemp flower extract used in the production of individual films.

### 2.9. Microbial Stability of the Freeze-Dried Fruit During Storage in the Tested Films Polysaccharides

**Figure 14:**
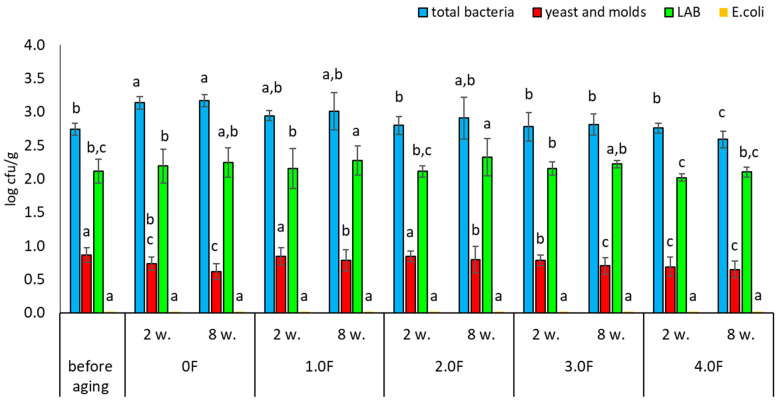
Microbial stability of raspberries during storage. The letters (a, b, c, …) next to the mean values indicate statistical significance of differences be-tween groups, determined by a post-hoc test (Tukey).

The microbiological quality of freeze-dried fruits, including raspberries, represents an important area of research in the context of food safety and maintaining high quality of the final product. Although freeze-drying is considered an effective preservation method, allowing the retention of nutritional value and a significant extension of shelf life, it does not guarantee the complete elimination of microbiological hazards. Contamination may be introduced at various stages of the technological process—from raw material preparation, through the drying process, to storage and packaging of the product. In the present study, the microbiological stability of freeze-dried raspberries and blueberries was analysed. After the drying process, the fruits were packaged in bioactive films containing *C. sativa* flower extract, characterized by antimicrobial and antioxidant properties [75]. The obtained results indicate that the type of film used had a measurable, though limited, effect on microbiological changes during fruit storage. The total bacterial count in raspberries after freeze-drying (before packaging) was 2.74 log cfu/g, whereas after 8 weeks of storage in the control film (0 F) it increased to 3.17 log cfu/g (Figure 14). Slightly lower values were observed in samples stored in the tested films, with the lowest level (2.59 log cfu/g) recorded for the 4.0 F film. Although these differences were statistically significant, their absolute magnitude was small, suggesting a moderate stabilizing effect. A similar trend was observed for lactic acid bacteria (LAB); in raspberries stored in the 4.0 F film, no significant changes in LAB counts were recorded compared to the initial values. The most noticeable changes were observed for yeasts and molds. In all stored raspberry samples, a decrease in their counts was noted after 2 and 8 weeks, with the greatest reduction (from 0.86 to 0.64 log cfu/g) recorded in samples stored in the 4.0 F film. However, it should be emphasized that the range of these changes was also limited. In addition, no presence of Escherichia coli was detected in raspberry samples throughout the entire storage period.

**Figure 15:**
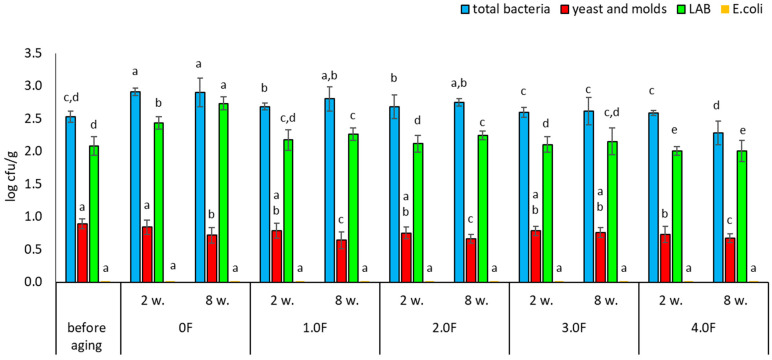
Microbial stability of blueberries during storage. The letters (a, b, c, …) next to the mean values indicate statistical significance of differences be-tween groups, determined by a post-hoc test (Tukey).

**Figure 16:**
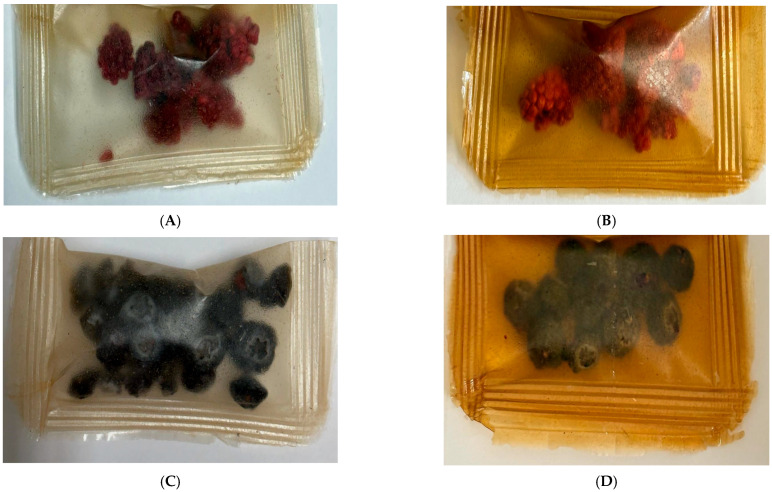
Images of raspberries (**A**,**B**) and blueberries (**C**,**D**) stored at 0 F after 8 weeks (**A**,**C**) and at 4.0 F after 8 weeks (**B**,**D**).

Very similar trends were observed for freeze-dried blueberries. Among the analysed variants, the 4.0 F film showed the highest potential for microbiological stabilization. The total bacterial count before storage was 2.52 log cfu/g, while after 8 weeks of storage in the 4.0 F film it decreased to 2.28 log cfu/g (Figure 15). A reduction in yeast and mould counts was also observed—from 0.89 to 0.67 log cfu/g after 8 weeks of storage (Figure 16). As in the case of raspberries, no growth of *E. coli* was detected in blueberry samples during the entire study period. Literature data indicate that dried fruits may contain a wide range of microorganisms, including bacteria such as Escherichia coli and filamentous fungi of the genera Aspergillus and Penicillium [76]. Among the microorganisms most frequently isolated from raspberries—both fresh and freeze-dried—are also yeasts, including Hanseniaspora uvarum and Saccharomyces cerevisiae [77,78]. The degree of microbiological contamination of freeze-dried fruits is strongly dependent on the quality of the raw material and the parameters of the technological process. Numerous studies indicate that microbial levels in such products typically range from 1.0 to 4.0 log cfu/g [79,80,81]. The results obtained in the present study fall within these ranges and confirm that although freeze-drying significantly reduces the number of microorganisms, it does not eliminate them completely, and some strains may exhibit increased resistance [81]. The use of antimicrobial active packaging currently represents one of the directions in the development of technologies aimed at extending the shelf life of freeze-dried fruits. These solutions are based on the use of bioactive substances that may limit microbial growth during product storage. Examples include polylactic acid (PLA)-based materials enriched with essential oils, which have been shown to inhibit the growth of yeasts and molds compared to conventional packaging [82]. Similarly, Espitia et al. (2012) demonstrated the effectiveness of packaging systems using essential oil sachets, which limit fungal growth without direct contact between the active substance and the product [83]. Modern solutions also include the use of nanocomposites, such as films containing silver nanoparticles combined with gelatine, which may inhibit microbial growth on fruit surfaces [84]. Zorić et al. (2015) emphasized that properly designed packaging materials can also limit moisture absorption and the loss of bioactive compounds, which is important for the quality of freeze-dried products [74]. The development of antimicrobial polymer packaging responds to the growing consumer demand for minimally processed food [74], and as highlighted by Díez-Pascual (2020), constitutes an important element of strategies ensuring food quality and safety [85]. Biopolymers such as chitosan exhibit natural antimicrobial properties, making them attractive components of active packaging systems [86]. At the same time, the development of nanoporous films enabling humidity control shows potential in limiting mould growth during the storage of fresh and processed fruits [87]. Based on the available knowledge, the present study represents one of the first investigations in which films containing bioactive compounds isolated from *C. sativa* flower extract were used to assess the microbiological stability of freeze-dried raspberries and blueberries—fruits that have rarely been analysed in this context during long-term storage.

### 2.10. Analysis of the Results of the Study

**Figure 17:**
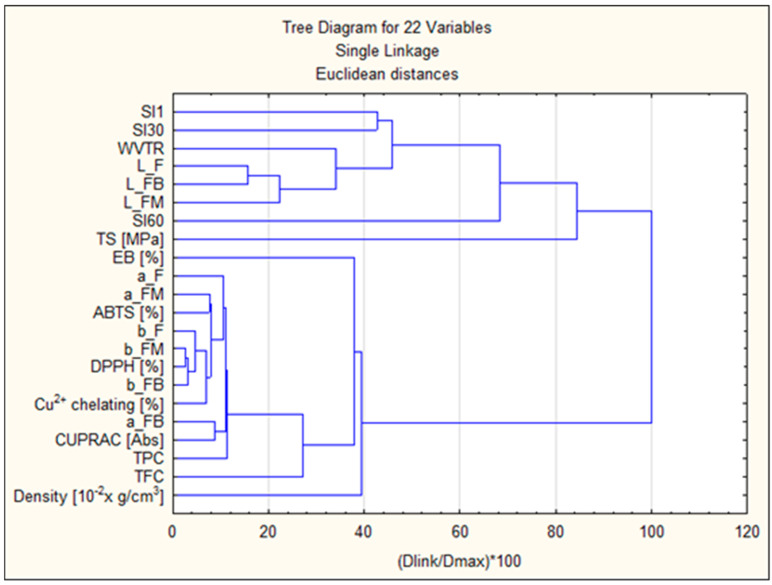
Cluster analysis diagram.

In order to conduct the cluster analysis (Figure 17), the indicated parameters were used: swelling coefficients after 1, 30, and 60 min (SI1, SI30, SI60), film colour parameters before fruit incubation and after 8 weeks of incubation (L_F, L_FB, L_FM, a_F, a_FB, a_FM, b_F, b_FB, b_FM), antioxidant parameters (ABTS [%], DPPH [%], Cu^2+^ chelating [%], CUPRAC [Abs], TPC [%], TFC [%]), strength parameters (TS [MPa], EB [%], WVTR) and Density [10^−2^ × g/cm^3^]. Clustering 1 included surface/barrier and mechanical parameters SI1, SI30, WVTR, L_F, L_FB, L_FM, SI60, TS [MPa], EB [%]. These target variables cluster together earlier than the others, confirming that mechanical properties and some brightness/L parameters are more similar within this group. Clustering 2 mainly included a_F, a_FM, ABTS [%], b_F, b_FM, DPPH [%], b_FB, Cu^2+^ chelating [%], a_FB, CUPRAC [Abs], TPC, TFC, Density. In this group, strong correlations were observed between antioxidant tests (DPPH, ABTS, CUPRAC, Cu^2+^ chelation) and measures of phenolic content (TPC, TFC) and colour parameters (a*/b*). This is evidence that colour/colour parameters are correlated with phenolic compound content and antioxidant activity. Strong correlations between TPC/TFC and values (CUPRAC, Cu^2+^ chelation) mean that increased polyphenol content is associated with higher antioxidant activity. Correlation between colour (* a, * b) and antioxidant tests ** suggests that colour changes may be an indicator of chemical changes (e.g., phenolic extracts/colourants affecting both colour and activity).

**Figure 18:**
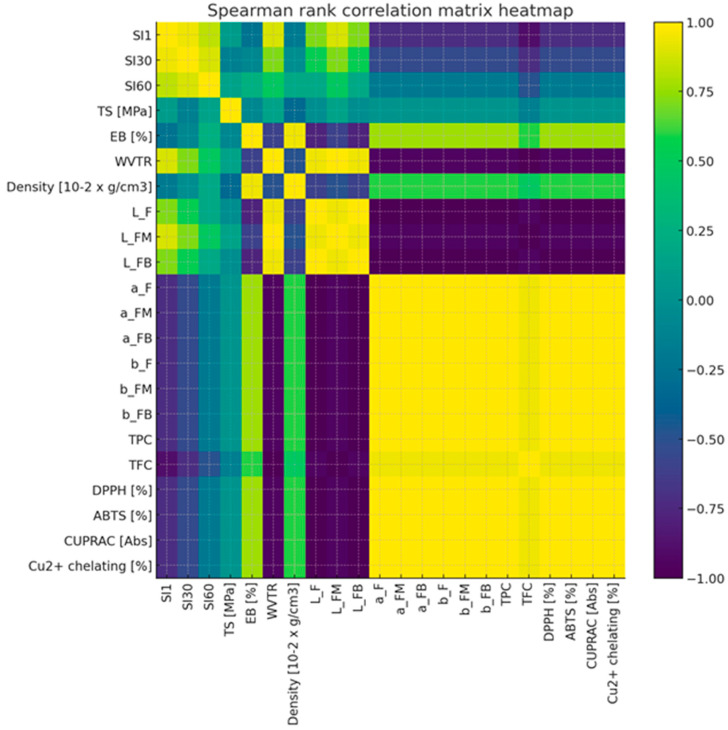
Spearman rank correlation matrix heatmap.

**Figure 19:**
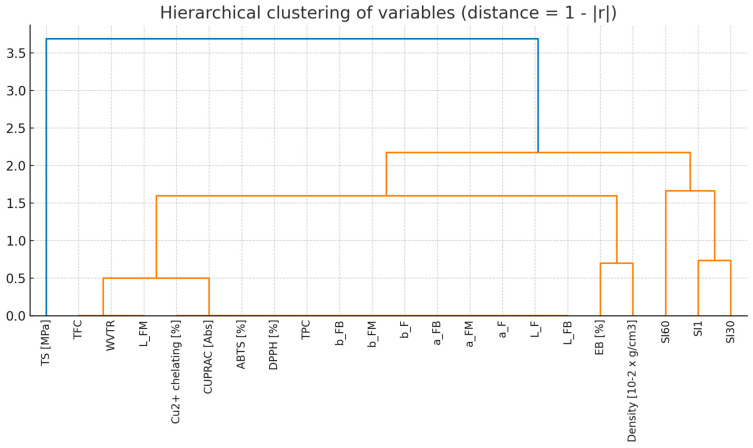
Hierarchical clustering of variables (distance—1 − |r|).

The Spearman’s rank correlation analysis was performed for 22 variables describing the properties of the tested samples. The correlation matrix (Figure 18) revealed a very high level of interdependence—as many as 67.5% of all attribute couples showed a strong correlation (|r| ≥ 0.7), and 35.1% of couples achieved a perfect correlation (|r| = 1.0). In particular, strong correlations were observed between colour indices (L*, a*, b*) and parameters describing antioxidant activity (TPC, DPPH, ABTS, CUPRAC, Cu^2+^ chelating capacity), which formed consistent blocks of variables. In contrast, film swelling indexes (SI1, SI30, SI60) and other physical parameters, such as density or elongation at break (EB), showed relatively weaker correlations with other groups of characteristics. Hierarchical clustering (Figure 19) (average linkage method, distance = 1 − |r|) was used to reduce the dimensionality. Based on the dendrogram (cut-off threshold: 0.3), 8 clusters of variables were identified. For each cluster, a representative variable was selected, which allowed the number of analysed features to be reduced from 22 to 8 while keeping most of the information contained in the original data set. The proposed set includes: WVTR, L_F, EB [%], density, SI1, SI30, SI60 and TS [MPa]. The cluster representatives for each item were: WVTR—represents the block: WVTR, L_FM, TFC; L_F—representing the block: L_F, L_FB, a_F, a_FM, a_FB, b_F, b_FM, b_FB, TPC, DPPH, ABTS, CUPRAC, Cu^2+^ chelating; while EB [%], Density [10^−2^ × g/cm^3^], SI1, SI30, SI60 and TS [MPa]—provided an independent indicator. This approach would minimise the problem of co-linearity, simplifies models and allows for a clearer interpretation of results.

## 3. Materials and Methods

### 3.1. Preparation of Extract from the Flowers of Cannabis sativa

Cannabis flowers were used to obtain the active ingredients. The powdered cannabis flowers were used to obtain a 50% ethanol extract in water with a concentration of 1 g/mL, as in the previously published articles [8,88].

### 3.2. Study of the Extract from the Flowers of Cannabis sativa

Analysis GC-MS of prepared extract was performed on a Bruker gas chromatograph with mass detection. The extract was obtained as follows: 4 mL of ethanol was added to 1.0 g of hemp flowers, heated for 5 min at boiling point in a heating flask, then cooled and filtered through a 0.20 µm syringe filter. The filtrate was used for GC-MS testing. The test was performed under the following parameters: electron energy 70 eV; ion source at 200 °C; silica column—VF-5 ms (30 m × 0.25 mm × 0.39); df = 0.25 μm; carrier gas—helium; gas flow rate—1 mL/min. The identification of compounds was based on a comparison of their retention times and mass spectra with NIST standards. The test parameters and conditions were the same as in the previous papers [8,88]. The quantification of selected flavonoids (isoquercetin, astragalin, rutin) and phenolic acids (ferulic and p-coumaric acids) in *C. sativa* flower extract was performed using high-performance liquid chromatography (HPLC) (Dionex Thermoline Fisher Scientific, Waltham, MA, USA) with Chromeleon software version 7.0, following the protocol established by Paczkowska-Walendowska et al. (2021) [89]. Separations were performed on a LiChrospher RP-18 column, 5 μm particle size, 250 mm × 4 mm (Merck, Darmstadt, Germany). Chromatographic separation was achieved using a gradient elution with mobile phase A consisting of 0.1% formic acid in water, and mobile phase B of acetonitrile. The gradient profile was as follows: 0–35 min, 2–20% B; 35–55 min, 20–70% B; followed by re-equilibration at 2% B from 55 to 60 min. The flow rate was maintained at 1.0 mL/min, the column temperature was set at 40 °C, and detection wavelengths were set at 360 nm for flavonoids, 240 nm for ferulic acid, and 265 nm for p-coumaric acid. Standard solutions were prepared using analytical-grade reference compounds dissolved in HPLC-grade methanol. Calibration standards included: isoquercetin (0.5 mg/mL), astragalin (0.01–1.0 mg/mL), rutin (0.5 mg/mL), ferulic acid (0.001–0.1 mg/mL), and p-coumaric acid (0.001–0.1 mg/mL). All solutions were filtered through 0.22 µm membrane filters prior to injection. Calibration curves were generated based on peak areas, and method validation parameters included linearity, intra- and inter-day precision, as well as determination of the limits of detection (LOD) and quantification (LOQ).

### 3.3. Preparation of Active Hydrocolloid-Based Films Polysaccharides

Citrus pectin (PC) and gellan gum (GG) were used to produce the film in a 1:1 ratio. Destilled water was the main medium. Ethanol extract was added to the solution in the following amounts: 0.5 [wt.%], 1.0 [wt.%], 2.0 [wt.%], 3.0 [wt.%] and 4.0 [wt.%]. The obtained solutions were put under magnetic stirring at 600 rpm for 85 min at 105 °C. The cast film samples were dried at 22 °C for 72 h in order to produce a thin film [90]. The reference sample was a sample without the addition of extract. The obtained film samples were identified as follows: 0, 0.5 F, 1.0 F, 2.0 F, 3.0 F, 4.0 F.

### 3.4. Testing of Developed Polysaccharide Films

All fabricated active hydrocolloid-based films polysaccharides were stored at a relative humidity of 50 ± 5 [%] and a temperature of 23 °C. The average thickness of the film was determined using a digital micrometer (Mitutoyo Digimatic IP65). FTIR analysis was performed using the same apparatus and method as in the study (Dobrucka et al., 2024, Szymanski et al. 2024) [8,88]. The colour test of the film and fruit was performed using an EnviSense NR60CP colorimeter, as in previous studies [8,88]. The water vapour transmission rate (WVTR) and mechanical test (TS, EB) was performed in the same way as in the study [8,88,90,91]. Tests of film swelling were performed using an Evo 40 scanning electron microscope (Zeiss, Oberkochen, Germany) and a Zeiss SteREO Discovery.V8 stereoscopic microscope.

### 3.5. Research on TPC, TFC, Antioxidant Activity of the Film and Extract (DPPH, ABTS, CUPRAC, Cu^2+^ Chelating)

#### 3.5.1. Determination of Total Phenolic Content (TPC)

The total phenolic content was determined using the method described by Studzińska-Sroka et al. (2024), with minor modifications [92]. Briefly, 25.0 μL of *C. sativa* flower extract (31.25 mg of dry weight/mL), film solution (10 mg film/mL), or gallic acid standard solution (6.25–100 μg/mL) was mixed with 200.0 μL of distilled water, 15.0 μL of Folin–Ciocalteu reagent, and 60.0 μL of 20% sodium carbonate solution in a 96-well plate. Results were expressed as milligrams of gallic acid equivalents (GAE) per gram of dry weight of plant material or per gram of film ± standard deviation (SD) [92].

#### 3.5.2. Determination of Total Flavonoid Content (TFC)

Total flavonoid content was assessed according to the procedure reported by Studzińska-Sroka et al. (2024), with slight modifications [93]. In brief, 100.0 μL of *C. sativa* flower extract (31.25 mg of dry weight/mL), film solution (10 mg film/mL), or standard was mixed with 100.0 μL of 2% aqueous aluminium chloride solution. The results were expressed as milligrams of quercetin equivalent (QE) per gram of dry weight of plant material or per gram of film ± standard deviation (SD) [93].

#### 3.5.3. DPPH Scavenging Activity

The antiradical activity was evaluated using the DPPH assay, as previously described (Studzińska-Sroka et al., 2024) [93]. In brief, 25.0 μL of *C. sativa* flower (concentration range: 0.98–31.25 mg of dry weight/mL) or film solution (10 mg film/mL) was mixed with 175.0 μL of DPPH solution (3.9 mg/50 mL methanol). Antiradical activity was expressed as IC_50_ (mg/mL) for the extract and as the percentage of radical scavenging activity (mean ± SD) for the film samples [93,94].

#### 3.5.4. ABTS Scavenging Activity

The antiradical activity was assessed using a modified ABTS assay, based on a previously reported method (Chanaj-Kaczmarek et al., 2015) [95]. In short, 10.0 μL of *C. sativa* flower (concentration range: 0.98–15.63 mg of dry weight/mL) or film solution (10 mg film/mL) was added to 200.0 μL of ABTS•^+^ solution (7 mM ABTS•^+^ generated by reaction with 2.45 mM potassium persulfate and diluted with water to an absorbance of ~0.77 at 734 nm). The antioxidant activity was expressed as IC_50_ (mg/mL) for the extract and as the percentage of radical scavenging activity (mean ± SD) for the film samples [95].

#### 3.5.5. CUPRAC Assay

The CUPRAC assay was performed following a previously published protocol with slight modifications (Studzińska-Sroka et al., 2024) [93]. A volume of 50.0 μL of the tested *C. sativa* flower extract (concentration range: 0.98–31.25 mg of dry weight/mL) or film solution (10 mg film/mL) was mixed with 150.0 μL of the CUPRAC reagent. Results were expressed as IC0.5 (mg/mL) for the extract and as the percentage of radical scavenging activity (mean ± SD) for the film samples [93].

#### 3.5.6. Determination of Cu^2+^ Chelating Activity

The Cu^2+^ chelating capacity of the samples was assessed according to the method described by Paczkowska-Walendowska et al. (2023), with slight modifications [96]. Briefly, 30.0 μL of *C. sativa* flower extract (0.98–15.63 mg of dry weight/mL) or film solution (10 mg film/mL) was mixed with 175.0 μL of sodium acetate buffer (50 mM, pH ~6) and 30.0 μL of CuSO_4_·5H_2_O (0.4 mM) in a 96-well plate. Results were expressed as IC_50_ (mg/mL) for the yellow dock root extract and as a percentage of chelating activity for the film formulations ± SD [96].

### 3.6. Dissolution Studies

Dissolution studies of the prepared films were carried out using a modified version of the method described by Paczkowska-Walendowska et al. (2023) [96]. Briefly, 40 mL of distilled water was added to a beaker, and film samples with a surface area of 4 cm^2^ (0.5 F, 1 F, 2 F, 3 F, 4 F) were immersed in the medium. At predetermined time intervals (0.5 h, 1.0 h, 2.0 h, 4.0 h, and 6.0 h), aliquots of the dissolution medium were collected, filtered through a 0.45 μm nylon membrane, and analysed for the content of isoquercetin, ferulic acid, and p-coumaric acid (compounds present in the extract at concentrations >1 mg/100 g DW) using a previously validated HPLC method [97].

### 3.7. Testing of Packaged Freeze-Dried Raspberry (Rubus idaeus L.) and Blueberry (Vaccinium corymbosum L.)

#### 3.7.1. Preparation of Freeze-Dried Fruits for Testing

Red raspberry (*Rubus idaeus* L.) and blueberry (*Vaccinium corymbosum* L.) fruits were purchased in retail stores in May 2025. The moisture content of the starting raw material was 85.7% and 82% per 100 g of fresh fruit, respectively. To determine the effect of packaging type on stored fruit, fresh fruit was freeze-dried using a Martin Christ Alpha 2–4 LDplus device (Martin Christ, Osterode am Harz, Germany). The samples were initially frozen at −80 °C for 24 h, then the freeze-drying process was carried out at −80 °C under a vacuum of 0.12 mbar in a drying chamber. The drying process was carried out at a temperature of −42 °C [98]. The freeze-drying process was conducted for 72 h. The moisture content of the raw material was 6% for raspberries, 8.5% for blueberries. The freeze-dried fruits prepared in this way were packed in foil with the addition of *C. sativa* flower extract and stored for 8 weeks at 21 °C. The freeze-dried raspberry and blueberry fruits were ground to homogenize the sample using a laboratory mill (Witko, Łódź, Poland). The samples were stored in sealed Falcon containers under refrigeration conditions (4 °C) until analysis.

#### 3.7.2. Extraction of Phenolic Compounds in Fruits

To prepare the extracts, 0.5 g of a homogeneous sample was weighed into a centrifuge tube, and 10 mL of water was added as an extraction agent. It was shaken for 60 min on a rocking shaker and centrifuged at 4125× *g* for 7 min. The supernatant (extract) was decanted and used for the following analyses. The procedure for preparing samples for analysis was developed based on our research [97].

#### 3.7.3. Determination of TPC in Fruits

The total phenolic content (TPC) was determined using the Folin–Ciocalteu reagent (Singleton and Rossi, 1965) and the methodology described by Gumienna et al. (2025) [97,98]. The following were added to the tubes: 0.3 mL of fruit extract, 0.05 mL of Folin–Ciocalteu reagent (2 N), (Sigma-Aldrich, Munich, Germany), then 0,5 mL of 2% aqueous Na_2_CO_3_ solution, 4150 mL water (Sigma-Aldrich, Munich, Germany) was added and water was added to the final volume of 10 mL. The samples were incubated for 30 min in the dark at room temperature. Absorbance was measured at 700 nm using a UV–Vis spectrophotometer Halo SB-10 (Dynamica Biogenet, Cambridge, UK). Total phenolic content was expressed as gallic acid equivalents (GAE) (Sigma-Aldrich, Munich, Germany) per gram of dry sample weight (g GAE/100 g d.m.) [97,98].

#### 3.7.4. Determination of TEAC in Fruits

Total antioxidant capacity (TEAC) was determined with the ABTS reagent (2,20-azinobis-(3-ethylbenzothiazoline-6-sulfonic acid) (Sigma-Aldrich, Munich, Germany) [Re et al., 1999] based on the method described by Gumienna et al. (2025) [60,97]. 3 mL of ABTS· solution, 0.03 mL of fruit extract. After 6 min of incubation in the dark, the absorbance was measured at 735 nm using a spectrophotometer (Biogenet, Józefów, Poland). The ABTS test results were expressed as the antioxidant capacity to scavenge ABTS radicals to Trolox (a water-soluble vitamin E analogue) and were given as g TE/100 g dm (Sigma-Aldrich, Munich, Germany) [60,98].

#### 3.7.5. Assessment of the Number of Microorganisms in Fruits

Under aseptic conditions, the films in which the fruits were stored were opened. Fragments were cut off from 3 selected fruits (raspberries and blueberries) to obtain 1 g of freeze-dried material. This was placed in a 250 cm^3^ Erlenmeyer flask, next 50 cm^3^ of physiological saline (0.85% NaCl) was poured over it and shaken for 15 min at 22 °C at 120 rpm. Then, sample plating was applied using selected growth media: nutrient broth agar to determine the total number of bacteria (Millipore, Darmstadt, Germany), MRS agar to determine lactic acid bacteria (BTL, Łódź, Poland), MacConkey Agar to determine Escherichia coli bacteria (Biomaxima, Lublin, Poland), and Chloramphenicol substrate (BTL, Łódź, Poland) to determine yeasts and moulds. Samples were incubated at 37 °C for 24–48 h. The results were expressed in log cfu/g.

### 3.8. Statistical Analyses

To verify the correlations between the samples, the values of the parameters (SI1, SI30, SI60, TS [MPa], EB [%], WVTR, Density [10^−2^ × g/cm^3^], L_F, L_FM, L_FB, a_F, a_FM, a_FB, b_F, b_FM, b_FB, TPC, TFC, DPPH [%], ABTS [%], CUPRAC [Abs], Cu^2+^ chelating [%]) were tabulated, and Spearman’s rank correlation test was conducted. In addition, a test comparing averages (ANOVA) was performed for all results. Correlation coefficients were considered statistically significant at *p* < 0.05. All calculations were performed using STATISTICA 13. The following thresholds were applied: uncorrelated for R = 0; weak correlation for 0 < R < 0.5; strong correlation for 0.5 ≤ R < 0.7; very strong correlation for 0.7 ≤ R < 0.9; nearly complete correlation for 0.9 ≤ R < 1; and complete correlation for R = 1. Hierarchical clustering (average linkage method, distance = 1 − |r|) was used to reduce dimensionality. Additionally, cluster analysis was performed using STATISTICA version 13.

## 4. Conclusions

In this study, hydrocolloid-based films enriched with *Cannabis sativa* flower extract were successfully developed for packaging freeze-dried raspberries and blueberries. The incorporation of the extract significantly improved the mechanical and barrier properties of the films, with the 4.0 F formulation exhibiting the highest elasticity and the lowest water vapor permeability. The results obtained are the effect of including the appropriate active compounds contained (which were evaluated using HPLC and GC) in the flower extract. Spearman rank correlation analysis was performed for 22 variables describing the properties of the tested samples and showed particularly strong correlations between color indices (L*, a*, b*) and parameters describing antioxidant activity (TPC, DPPH, ABTS, CUPRAC, Cu^2+^ chelating capacity), which formed coherent blocks of variables. The research showed a significant impact of the type of film used on the microbiological stability of stored raspberries and blueberries. These results demonstrate the potential of polysaccharide-based films containing *C. sativa* extract as active packaging, capable of extending the shelf life and maintaining the quality of freeze-dried fruits.

## Figures and Tables

**Table 1:** Result of analysis of *C. sativa* flower extract.

Peak RT (min)	Area	Quantity (%)	Compound Name	Formula
5.724	2.03 × 10^6^	0.024	β-phellandrene	C_10_H_16_
13.464	4.09 × 10^7^	0.482	[1R-(1R*,4Z,9S*)]-4,11,11-trimethyl-8-methylene-bicyclo[7.2.0]undec-4-ene	C_15_H_24_
13.582	4.04 × 10^6^	0.048	7-epi-trans-sesquisabinene hydrate	C_15_H_26_O
13.929	1.73 × 10^7^	0.203	humulene	C_15_H_24_
14.386	5.23 × 10^6^	0.062	α-acorenol	C_15_H_26_O
14.518	9.04 × 10^6^	0.106	ß-bisabolene	C_15_H_24_
14.586	4.87 × 10^6^	0.057	dihydro-ß-agarofuran	C_15_H_26_O
14.702	5.87 × 10^6^	0.069	4-epi-cubedol	C_15_H_26_O
14.944	2.10 × 10^7^	0.247	alloaromadendrene	C_15_H_24_
15.007	1.70 × 10^7^	0.200	selina-3,7(11)-diene	C_15_H_24_
15.517	1.46 × 10^7^	0.172	caryophyllene oxide	C_15_H_24_O
15.659	6,12 × 10^7^	0.720	guaiol	C_15_H_26_O
15.840	1.15 × 10^7^	0.136	6-epi-shyobunol	C_15_H_26_O
16.026	8.47 × 10^7^	0.997	8-epi-.gama.-eudesmol	C_15_H_26_O
16.363	7.81 × 10^7^	0.919	ß-eudesmol	C_15_H_26_O
16.443	6.82 × 10^7^	0.803	α-eudesmol	C_15_H_26_O
16.624	1.11 × 10^8^	1.310	α-bisabolol	**C_15_H_26_O**
18.120	2.38 × 10^7^	0.280	3,7,11,15-tetramethyl-2-hexadecen-1-ol	C_20_H_40_O
18.188	5.74 × 10^6^	0.068	dihydro-ß-agarofuran	C_15_H_26_O
18.369	8.32 × 10^6^	0.098	2-cis-9-octadecenyloxyethanol	C_20_H_40_O_2_
18.563	1.36 × 10^7^	0.160	3,7,11,15-tetramethyl-2-hexadecen-1-ol	C_20_H_40_O
19.304	2.27 × 10^6^	0.027	1-heptatriacotanol	C_37_H_76_O
21.947	1.29 × 10^7^	0.152	olean-12-ene-3,28-diol, (3ß)-	C_30_H_50_O_2_
22.000	3.32 × 10^6^	0.039	erythrodiol	C_30_H_50_O_2_
22.442	7.68 × 10^7^	0.904	Δ8-tetrahydrocannabinol	C_21_H_30_O_2_
23.857	7.39 × 10^9^	86.951	cannabidiol	**C_21_H_30_O_2_**
24.410	1.72 × 10^8^	2.024	Δ^9^-tetrahydrocannabinol	**C_21_H_30_O_2_**
25.112	2.24 × 10^8^	2.637	8-ß hydroxy-Δ 9-tetrahydrocannabinol	**C_21_H_30_O_3_**
26.637	9.02 × 10^6^	0.106	2-(7-heptadecynyloxy)tetrahydro-2H-pyran	C_22_H_40_O_2_

## Data Availability

The original contributions presented in this study are included in the article. Further inquiries can be directed to the corresponding authors.
